# Exercise-induced cardiac troponin release: do we need to worry?

**DOI:** 10.1093/ehjacc/zuag012

**Published:** 2026-01-21

**Authors:** Thijs M H Eijsvogels, Alma M A Mingels, Johannes Mair, Nicholas L Mills, Bertil Lindahl

**Affiliations:** Department of Medical BioSciences, Radboud University Medical Center, Nijmegen, The Netherlands; Department of Clinical Chemistry, Central Diagnostic Laboratory, Maastricht University Medical Center, Maastricht, The Netherlands; CARIM School for Cardiovascular Diseases, Maastricht University Medical Center, Maastricht, The Netherlands; Department of Internal Medicine III—Cardiology and Angiology, Medical University of Innsbruck, Innsbruck, Austria; BHF Centre for Cardiovascular Science, University of Edinburgh, SU.226 Chancellor’s Building, Royal Infirmary of Edinburgh, 49 Little France Crescent, Edinburgh EH16 4SU, UK; Department of Medical Sciences, Uppsala University, Uppsala, Sweden

**Keywords:** Athletes, Sports cardiology, Biomarkers, Acute coronary syndrome, Cardiovascular

## Abstract

**Graphical Abstract:**
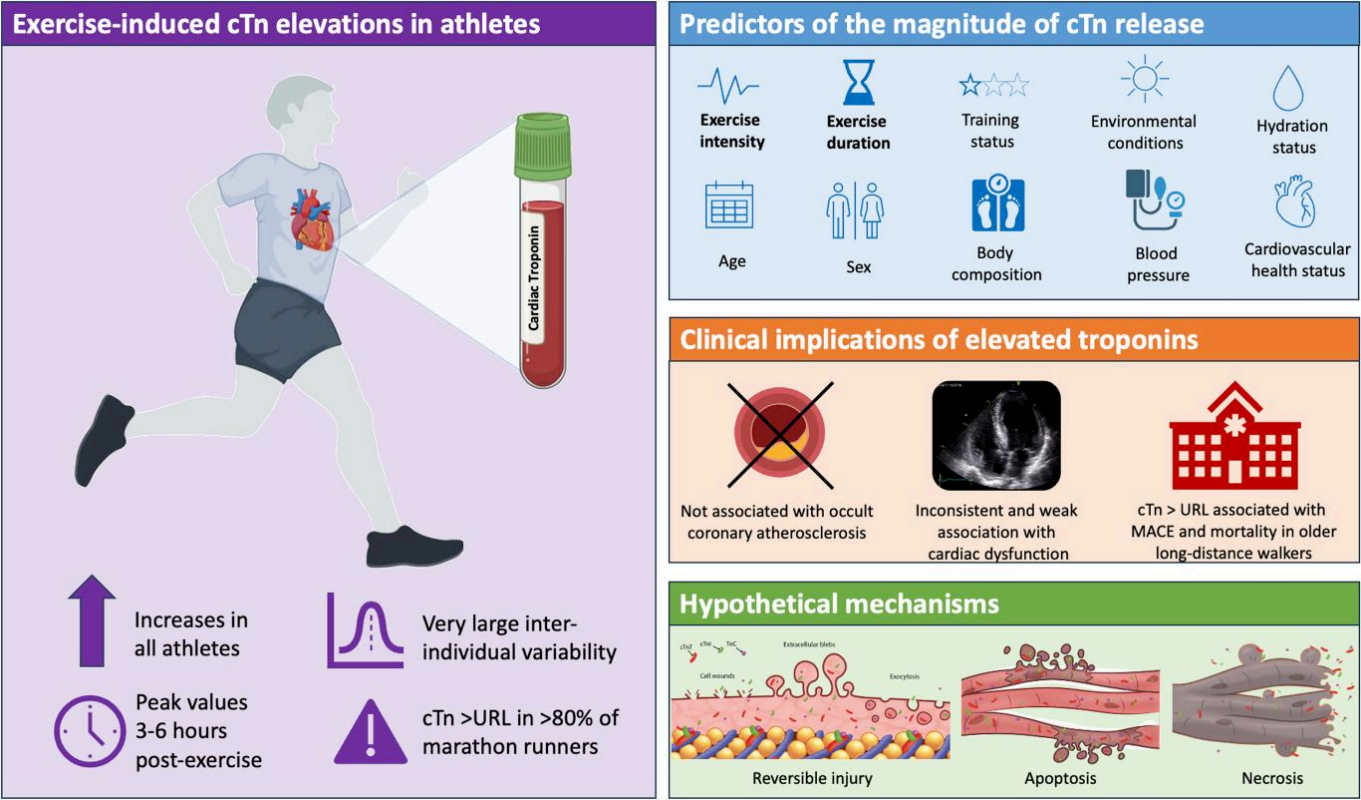

Cardiac troponins (cTn), either T (cTnT) or I (cTnI), are the preferred biomarkers for the diagnosis of myocardial infarction because of their excellent diagnostic performance. The detection of a typical rise and fall pattern is integrated in accelerated diagnostic pathways using high-sensitivity assays to triage patients with chest pain in the Emergency Department. The interpretation of elevated cTn concentrations in athletes can, however, be complicated as recent engagement in endurance exercise also produces transient cTn elevations. For example, marathon runners have a median hs-cTnT and hs-cTnI concentration of 30–50 ng/L post-race, with >80% of athletes having concentrations that exceed the diagnostic cutoffs.^1^ This raises the question whether exercise-induced cTn elevations are a physiological or pathophysiological phenomenon, or maybe a combination thereof.

cTn concentrations typically peak at 3–6 h after exercise and show considerable inter-individual variation between athletes. Previous studies have identified personal and exercise-related factors that are associated with the magnitude of exercise-induced cTn elevations, including age, sex, body composition, cardiovascular health status, blood pressure, training experience, exercise intensity and exercise duration.^1^ The intensity and duration of exercise are the strongest predictors, reflecting the magnitude of the exercise-induced workload of the heart. Nevertheless, the predictive value of these joint factors together remained low, only explaining around a third of post-exercise cTn concentrations.

There is ongoing debate as to the clinical relevance of exercise-induced cTn elevations. Data from population-based studies have shown that minor cTn elevations have predictive value for adverse health outcomes. A higher post-exercise cTn concentration may, therefore, be indicative of subclinical disease and/or cardiac vulnerability. Recently, the TREAT study showed no differences in coronary artery disease between athletes with very high vs. very low post-exercise cTn concentrations.^2^ Other studies reported inconsistent but weak associations between post-exercise cTn concentrations and cardiac dysfunction.^1^ Finally, a prospective study among older long-distance walkers found that post-exercise cTn concentrations above the upper reference limit (URL) were independently predictive of the composite outcome of major adverse cardiovascular events and mortality (Hazard Ratio: 2.5, 95% confidence interval: 1.3–4.8).^3^ The high age [61 (54–69) years] and prevalence of cardiovascular risk factors and diseases (40%) of the study population are, however, different from the typical endurance athlete, highlighting the need for other studies to confirm or challenge this observation.

It is thought that the mechanisms of cTn release can be due to (i) reversible injury attributable to cell wounds, cytoplasmic blebbing, or extracellular vesicle release; (ii) apoptosis; and (iii) myocardial necrosis.^4^ Only few studies explored the potential cTn release mechanisms in athletes. In one study, exercise led to compromised cardiomyocyte integrity as shown by cardiac magnetic resonance, and the magnitude of this effect was positively associated with post-exercise cTn concentrations.^5^ These findings suggest that degraded cTn forms may escape from the cytosol into the circulation,^6^ possibly reflecting a form of reversible injury. Nevertheless, the presence of some irreversible injury cannot be ruled out as contemporary MRI protocols have insufficient spatial resolution to quantify acute micro-injury. Further mechanistic studies are warranted.

Whether athletes and physicians ‘can run away’ from elevated post-exercise cTn concentrations depends on the clinical context and whether this is accompanied by possible cardiac symptoms. The general observation that endurance exercise produces cTn elevations in all athletes and that it might be caused by leakage of small cTn forms into the circulation, offers some reassurance in those without symptoms. However, prospective data on cTn and clinical outcomes in patients and in the general population consistently show positive associations between cTn concentrations and the risk of adverse health outcomes. Future exercise studies, especially in young and low-risk athletes with long-term follow-up on health outcomes, should point out what exactly to do in whom.

## Data Availability

No data were generated or analysed for this manuscript.

## Appendix

**Members of the Study Group on Biomarkers of the ESC Association for Acute Cardiovascular Care:**

Bertil Lindahl, MD^1^; Jasper Boeddinghaus, MD^2^; Louise Cullen, MD, PhD^3^; Lori Daniels, MD^4^; Ola Hammarsten, MD, PhD^5^; Kurt Huber, MD^6^; Evangelos Giannitsis, MD^7^; Allan S. Jaffe, MD^8^; Dorien M Kimenai, PhD^9^; Konstantin A. Krychtiuk, MD, PhD^10^; Martin Möckel, MD^11^; Christian Mueller, MD^2^; Matthias Thielmann, MD^12^; Kristian Thygesen, MD^13^; Johannes Mair, MD^14^; Nicholas L Mills, MD, PhD^9^

^1^ Department of Medical Sciences, University of Uppsala, Uppsala, Sweden

^2^ Cardiovascular Research Institute Basel (CRIB) and Department of Cardiology, University Hospital Basel, University of Basel, Switzerland

^3^ Emergency and Trauma Centre, Royal Brisbane and Women’s Hospital, Brisbane, Australia

^4^ Department of Medicine, Sulpizio Cardiovascular Center, University of California, San Diego; La Jolla, CA, United States of America

^5^ Department of Clinical Chemistry and Transfusion Medicine, University of Gothenburg, Gothenburg, Sweden

^6^ Department of Medicine, Cardiology and Intensive Care Medicine, Wilhelminenhospital, and Sigmund Freud University, Medical School, Vienna, Austria

^7^ Department of Cardiology, University Heidelberg, Germany

^8^ Departments of Cardiology and Laboratory Medicine and Pathology, Mayo Clinic, Rochester Minnesota, United States of America

^9^ BHF Centre for Cardiovascular Science, University of Edinburgh, Edinburgh, United Kingdom

^10^ Department of Internal Medicine II, Division of Cardiology, Medical University of Vienna, Vienna, Austria

^11^ Division of Emergency Medicine, Charité-Universitätsmedizin Berlin, Germany

^12^ Klinik für Thorax-und Kardiovaskuläre Chirurgie, Westdeutsches Herz- und Gefäßzentrum Essen, University of Duisburg-Essen, Germany

^13^ Department of Cardiology, Aarhus University Hospital, Denmark

^14^ Department of Internal Medicine III - Cardiology and Angiology, Innsbruck Medical University, Innsbruck, Austria

## References

[1] Aengevaeren VL, Baggish AL, Chung EH, George K, Kleiven Ø, Mingels AMA, et al Exercise-induced cardiac troponin elevations: from underlying mechanisms to clinical relevance. Circulation 2021;144:1955–1972. 34898243 10.1161/CIRCULATIONAHA.121.056208 PMC8663527

[2] Janssen SLJE, van Everdingen WM, Saalmink WBJ, Lamers SK, Vroemen WHM, Denessen EJS, et al Relationship between exercise-induced cardiac troponin elevations and occult coronary atherosclerosis in middle-aged athletes. J Am Coll Cardiol 2025;85:2370–2382. 40533126 10.1016/j.jacc.2025.04.047

[3] Aengevaeren VL, Hopman MTE, Thompson PD, Bakker EA, George KP, Thijssen DHJ, et al Exercise-induced cardiac troponin I increase and incident mortality and cardiovascular events. Circulation 2019;140:804–814. 31401842 10.1161/CIRCULATIONAHA.119.041627

[4] Mair J, Lindahl B, Hammarsten O, Müller C, Giannitsis E, Huber K, et al How is cardiac troponin released from injured myocardium? Eur Heart J Acute Cardiovasc Care 2018;7:553–560. 29278915 10.1177/2048872617748553

[5] Aengevaeren VL, Froeling M, Hooijmans MT, Monte JR, van den Berg-Faay S, Hopman MTE, et al Myocardial injury and compromised cardiomyocyte integrity following a marathon run. JACC Cardiovasc Imaging 2020;13:1445–1447. 32199849 10.1016/j.jcmg.2019.12.020

[6] Vroemen WHM, Mezger STP, Masotti S, Clerico A, Bekers O, de Boer D, et al Cardiac troponin T: only small molecules in recreational runners after marathon completion. J Appl Lab Med 2019;3:909–911. 31639766 10.1373/jalm.2018.027144

